# An *in vitro* method to test the safety and efficacy of low-level laser therapy (LLLT) in the healing of a canine skin model

**DOI:** 10.1186/s12917-016-0689-5

**Published:** 2016-04-08

**Authors:** Dominique Gagnon, Thomas W.G. Gibson, Ameet Singh, Alex R. zur Linden, Jaimie E. Kazienko, Jonathan LaMarre

**Affiliations:** Department of Clinical Studies, Ontario Veterinary College, University of Guelph, 50 Stone Road East, Guelph, ON N1G 2 W1 Canada; Department of Biomedical Sciences, Ontario Veterinary College, University of Guelph, 50 Stone Road East, Guelph, ON N1G 2 W1 Canada

**Keywords:** Canine epidermal keratinocytes progenitors, Low-level laser therapy, Proliferation assay, Scratch migration assay, Wound healing

## Abstract

**Background:**

Low-level laser therapy (LLLT) has been used clinically as a treatment modality for a variety of medical conditions including wound-healing processes. It is an attractive and emerging method to enhance wound healing and improve clinical outcomes both in human and veterinary medicine. Despite the fact that the use of LLLT continues to gain in popularity, there is no universally accepted theory that defends all its cellular effects and beneficial biological processes in tissue repair. The present study was designed to evaluate the effect of LLLT on cellular migration and proliferation of cultured canine epidermal keratinocytes (CPEK) in an in vitro wound healing model.

**Results:**

Keratinocyte migration and proliferation were assessed using a scratch migration assay and a proliferation assay, respectively. Fifteen independent replicates were performed for each assay. Canine epidermal keratinocyte cells exposed to LLLT with 0.1, 0.2, and 1.2 J/cm^2^ migrated significantly more rapidly (*p* < 0.03) and showed significantly higher rates of proliferation (*p* < 0.0001) compared to non-irradiated cells cultured in the same medium and cells exposed to the higher energy dose of 10 J/cm^2^. Irradiation with 10 J/cm^2^ was characterized by decreased cellular migration and proliferation. These results revealed that LLLT has a measurable, dose-dependent effect on two different aspects of keratinocyte biology in vitro.

**Conclusion:**

In this in vitro wound-healing model, LLLT increased cellular migration and proliferation at doses of 0.1, 0.2, and 1.2 J/cm^2^ while exposure to 10 J/cm^2^ decreased cellular migration and proliferation. These data suggest that the beneficial effects of LLLT in vivo may be due, in part, to effects on keratinocyte behavior.

## Background

The healing of skin wounds represents an ongoing challenge in both human and veterinary medicine [1, 2]. The care and management of both acute and chronic cutaneous wounds can be time consuming and may have significant health and economic consequences [2]. An emerging treatment modality involves the use of laser therapy, further described as low-level laser therapy (LLLT), which has been postulated to accelerate healing of damaged tissues and improve clinical outcomes in several independent research [3, 4]. Despite the positive results demonstrated in previous reports, the effectiveness and underlying mechanisms of this treatment modality remain largely speculative and unresolved [4, 5].

Wound healing was one of the first clinical applications of LLLT following its discovery by Endre Mester in 1967 [6, 7]. It has been suggested that the use of this treatment modality on cutaneous wounds provides several benefits, including pain control, reduction of inflammation, modulation of the immune system, and acceleration of wound healing [6–9]. In recent years, the medical applications of LLLT in human medicine have expanded to include the treatment of many conditions such as neurological processes, musculoskeletal injuries, soft tissue injury, abscesses, dental disease, and various dermatologic ailments [9, 10].

Low-level laser therapy causes low or imperceptible temperature changes to the treatment area [11]. At the cellular level, the effect of LLLT is thought to be secondary to the absorption of red and near-infrared light by cytochrome c oxidase, improving electron transport, production of adenosine triphosphate (ATP), release of nitric oxide, and the modulation of reactive oxygen species [10–12]. Although LLLT is believed to result in increased cellular metabolism via activation of the cellular respiratory chain [12, 13], other mechanisms of action have also been shown in recent literature which were associated to the effectiveness of this treatment modality [14–16]. Low-level laser therapy as a treatment modality still remains questionable due to an incomplete understanding of the specific underlying mechanism(s) responsible for the observed beneficial effects, and the large number of different irradiation protocols found in the literature, making comparisons between studies challenging [4, 5, 12]. For these reasons, LLLT remains an alternative treatment and its use is largely empirical [9, 12].

Numerous reports in the literature have shown beneficial results of LLLT on the rate and extent of wound healing using various models [3–13]. The effect of LLLT on in vitro cellular migration and proliferation of canine skin culture models has not been studied to date. The objective of this study was to evaluate both migration and proliferation of canine epidermal keratinocytes after different exposures/doses of LLLT in an in vitro wound-healing model.

## Methods

### Cell culture

Canine epidermal keratinocyte progenitors (CPEK) were purchased from CELLnTEC Advance Cell Systems^1^. These cells are cryo-preserved after isolation from normal tissue and have not undergone any transformation events. They were delivered from the manufacturer frozen in 1 mL vials. The CnT-09 medium, provided with the cell lines, was used for all CPEK canine epidermal cell cultures. CnT-09 is a liquid medium package including both basal medium (CnT-BM.2, 500 mL) and separate supplements (A [10 % fetal bovine serum (FBS), 50 mL) and B [L-glutamine, 5 mL]). The supplements can be used at different concentrations, depending on the volume added to the basal medium. For this study, a concentration of 10 % and 1 % (serum-deprived medium) medium were used.

All canine epidermal keratinocyte cells were cultured in 10 mL of 10 % serum medium (CnT-09) in 75 cm^3^ tissue culture flasks^2^ and incubated at 37 °C in an atmosphere of 5 % CO_2_ and 95 % air and passaged between 50 % and 90 % confluence. The medium was changed every forty-eight hours and replaced in the humidified incubator until an adequate numbers of cells were obtained for each experiment. For the scratch migration and proliferation assays, the cells were grown to approximately 90 % confluency and 40 % confluency, respectively. Third- to fifth-passage cells were used for all experiments.

### Study protocols

For both the scratch migration assay and proliferation assay, cells were trypsinized and plated on sterile 6-well tissue culture plates^b^. This process is known as Corning. For the scratch migration assay, the cells were seeded at 1 x 10^6^ cells per well in 2 ml keratinocyte growth medium CnT-09. For the proliferation assay, cells were seeded at a concentration of 4 × 10^4^ cells per well. For both experiments, plates were incubated for twenty-four hours under the conditions previously mentioned. Following this incubation, the medium of each well was replaced with 2 mL of low serum (1 %) medium, and incubated for an additional twelve hours. The medium of each well was then removed and replaced with phosphate buffered saline (PBS)^3^. For the scratch migration assay, a sterile p200 pipette tip^4^ was used to make a linear disruption of the monolayer of cells, simulating a wound-healing model. The scratch was created vertically in the middle of each well. The PBS was removed. The detached cells secondary to the creation of the scratch were carefully removed by rinsing each well with 1 mL of PBS. Formation of the in vitro wound was confirmed by inverted light microscopy^5^. To ensure that the same field was identified during subsequent image acquisition, two vertical lines on each side of the scratch and one horizontal line, separating the wound in half, were placed with an indelible marker on the outside bottom of each well. These markings served as reference points for photographic documentation.

### Experimental design

A randomized, blinded, and controlled study design was used to evaluate the effect of LLLT delivered at different doses over time on canine skin culture. Cellular migration and proliferation were determined using the scratch migration assay and proliferation assay, respectively. Six groups—composed of two different controls (positive and negative) and four LLLT treatments—were compared for the scratch migration assay. Five groups—composed of one control (negative) and the same four-LLLT treatments—were evaluated in the proliferation assay. Control groups were not exposed to LLLT. The control positive group was maintained in serum-rich medium, whereas the control negative group was maintained in serum-deprived medium. The LLLT (treatment) groups were exposed to different energy densities and were maintained in serum-deprived medium. A summary of the specific laser parameters used for each group is provided (Table 1). The energy density (J/cm^2^) for all treatment groups was calculated by multiplying the exposure time (s) by the power output of the laser, divided by the surface area (cm^2^). The surface area exposed to LLLT was identical to the surface area of a culture well of a 6-well plate (9.62 cm^2^).

**Table 1:** Low-level laser therapy protocols for all groups for the scratch migration assay and proliferation assay

Groups	Medium concentration	Distance	Spot size	Time	Power	Energy density
	(%)	(cm)	(cm^2^)	(s)	(W)	(J/cm^2^)
^a^Control positive	10	5	9.62	2	0	0
Control negative	1	5	9.62	2	0	0
Treatment 1	1	5	9.62	2	0.5	0.1
Treatment 2	1	5	9.62	2	1	0.2
Treatment 3	1	5	9.62	4	3	1.2
Treatment 4	1	5	9.62	8	12	10

^a^This group was not included in the proliferation assay

$$ \mathrm{Surface}\ \mathrm{area}\ \left({\mathrm{cm}}^2\right) = \pi {\mathrm{r}}^2 $$

$$ \mathrm{Irradiance}\ \left(\mathrm{J}/{\mathrm{cm}}^2\right) = \frac{\mathrm{Time}\ \left(\mathrm{s}\right)\ \mathrm{x}\ \mathrm{Power}\ \left(\mathrm{W}\right)}{\mathrm{Surface}\ \mathrm{area}\ \left({\mathrm{cm}}^2\right)} $$

Randomization of each group on the plate was achieved using a random number table provided in a statistics textbook [17]. The smallest number obtained represented the first well position, and then in ascending order, each group was assigned a position commencing with control positive, control negative, treatment 1, treatment 2, treatment 3, and treatment 4. Each experiment was run in quintuplicate using cells between the third- and fifth-passage. For the scratch migration assay, three plates, using each of the cell passage, represented one experiment. Specific to the proliferation assay, three plates were made per passage to allow cellular proliferation quantification over time. A total of fifteen independent replicates were therefore used for both assays.

### Low-level laser therapy

Low-level laser therapy was carried out using a high-power Helium-Neon (He-Ne) Class IV laser system^6^. Laser safety guidelines were followed as recommended by the manufacturer. Cells cultured in 6-well plates as described were irradiated using the large conical laser head provided. The handpiece was suspended from a custom-made stand holder and LLLT was carried at a fixed distance of five centimeters by measuring the distance between the laser head and the plate. This distance was specifically chosen as it provided uniform irradiation of the surface of each well. Low-level laser therapy was performed perpendicular to the bottom of the culture well, once, using continuous emission at a wavelength of 650 nm. It was performed in the same manner each time. The treated groups (treatment 1 to 4) were irradiated with one of the four-energy densities tested, as described above. The wells assigned to control groups (positive and negative controls) were sham-irradiated; they were maintained under the laser head for the minimum irradiation time used in the LLLT groups, without activating the laser source. During LLLT, the non-irradiated wells were covered using a clean, double-folded, white, commercial paper towel to prevent incidental irradiation. Following LLLT, 2 mL of serum-rich medium (10 %) was added to the well of the control positive, and 2 ml of serum-deprived medium (1 %) was added to the control negative and all four-treatment groups.

### Scratch migration assay

**Fig. 1:**
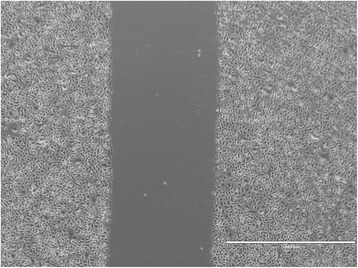
Representative scratch migration assay using cultured canine epidermal keratinocyte progenitors. Images were obtained from a motorized inverted microscope until complete closure of the scratch wound created in the cell monolayer was identified

**Fig. 2:**
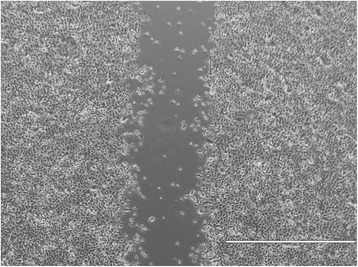
Representative scratch migration assay using cultured canine epidermal keratinocyte progenitors. Images were obtained from a motorized inverted microscope until complete closure of the scratch wound created in the cell monolayer was identified

**Fig. 3:**
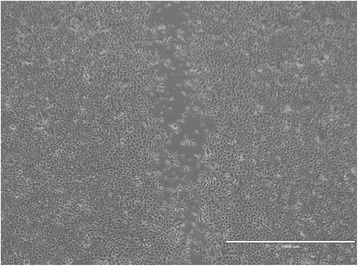
Representative scratch migration assay using cultured canine epidermal keratinocyte progenitors. Images were obtained from a motorized inverted microscope until complete closure of the scratch wound created in the cell monolayer was identified

**Fig. 4:**
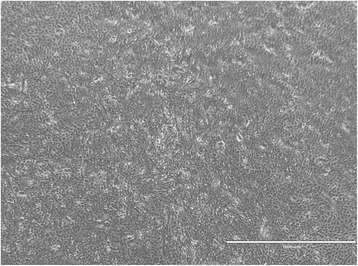
Representative scratch migration assay using cultured canine epidermal keratinocyte progenitors. Images were obtained from a motorized inverted microscope until complete closure of the scratch wound created in the cell monolayer was identified

A scratch was created in a cell monolayer as described above, which simulated a wound. The change in the wound surface area was compared among groups over time (Figs. 1, 2, 3, 4). Digital photographic images were obtained at the 0-, 12-, 24-, 36- and 48-h time points (or until complete closure of the scratch wound was observed) using a motorized inverted microscope^7^. Between each time point, the plates were incubated under the conditions described above. Following the acquisition of all images, the surface area of each scratch was measured and outlined by two independent observers (blinded to the treatment group) using the most recent Adobe Photoshop CC software^8^. The surface area of each wounded region of the cell monolayer was then transformed into a square of equal surface area, and the linear mean length of each square was compared among groups over time. The rate of closure was quantified and compared between all groups for statistical analysis.

### Proliferation assay

To evaluate and compare cellular proliferation among all groups over time, the ready-to-use cell proliferation reagent water-soluble tetrazolium (WST-1)^9^ was used. At the time of the experiment, 1.5 mL of medium was removed from each well, leaving a volume of 0.5 mL over the seeded cells. Fifty microliters of the cell proliferation reagent WST-1 was then added to each well of the 6-well acrylic plate, gently mixed with the medium for 30 s, and the plate was incubated for fifteen minutes under the conditions previously described. Three 100 μL aliquots from each well were transferred to a sterile 96-well spectrophotometer plate^10^. The quantity of formazan dye produced by cellular metabolism was quantified by measuring its absorbance at a wavelength at 450 nm using a microplate reader^11^ at 0-, 24- and 48-h time points. The mean absorbance obtained at each time point was compared between all groups for statistical significance.

The Department of Biomedical Sciences of the Ontario Veterinary College, University of Guelph, approved the study prior to commencement.

## Statistical analysis

For statistical analysis, the effect of LLLT delivered at various doses over time on canine skin culture was evaluated. For both the scratch migration assay and proliferation assay, a statistical program (SAS 9.2)^12^ was used to fit a general linear mixed model.

The design was a two-factor factorial in a randomized complete block (RCBD) design with fixed-effect factors treatment and time. To accommodate time being a repeated measure in the scratch migration assay, the following correlation structures (offered by SAS) were attempted: ar(1), arh(1), toep, toep(2), toep(3), toeph, toeph(2), toeph(3), un, un(2), and un(3). The random blocking effect was plates nested within cohorts. In the proliferation assay, time was not a repeated measure as destructive sampling occurred over time. To accommodate subsampling, a random effect of treatment by time by plates nested within cohorts was included in that model. Among the error structures that converged, the one with the smallest Akaike Information Criterion (AIC) was chosen. All terms up to the level of a 2-way interaction were considered. However, if terms were not significant, they were removed from the model. Differences were considered significant at *P* ≤ 0.05. To assess the ANOVA assumptions, comprehensive residual analyses were performed. The assumption of normality was formally tested by use of Shapiro-Wilk, Kolmogorov-Smirnov, Cramér-von Mises, and Anderson-Darling tests. Residuals were plotted against the predicted values and explanatory variables were used in the models to show outliers, bimodal distributions, the need for data transformations, or other issues that should be addressed.

## Results

### Scratch migration assay

A total of fifteen experiments were included in the study. To effectively meet the ANOVA assumptions of the statistical analysis, a square-root transformation was applied to all data as well as accommodating unequal variance in time (the error structure chosen was arh [1]). The results obtained at the 48- and 60-h time points were removed from the analysis as most of the groups tested were closed, except for the vast majority of cells irradiated with a higher dose of 10 J/cm^2^. No apparent outliers were identified in the analysis. There was no significant inter-observer variability observed for each surface area measured; all of the effects involving the observers had a *p* > 0.05.

**Fig. 5:**
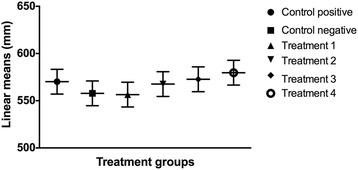
Effect of LLLT on wound size immediately after irradiation. Linear mean length with the confidence interval for each wounded area is presented. There was no significant difference noted among groups at the beginning of the experiment (*p* > 0.05). Significance *p* < 0.05

**Fig. 6:**
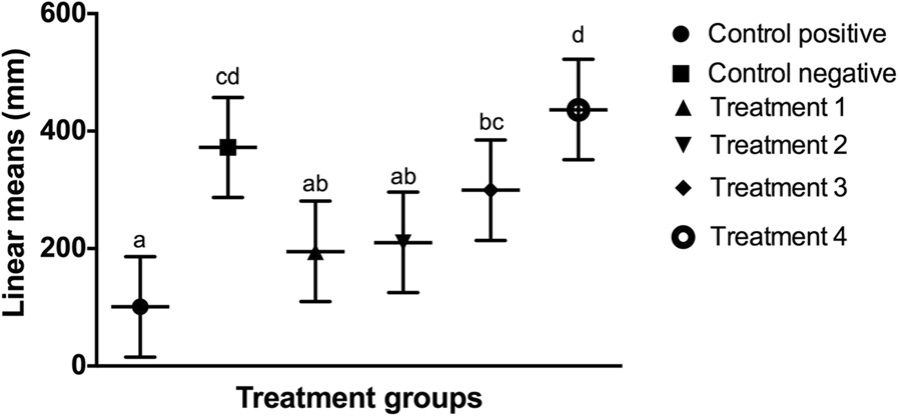
Effect of LLLT on wound size 12 h after irradiation. Linear mean length with the confidence interval for each wounded area is presented. There was a significant reduction in the linear mean length of the control positive (*p* < 0.0001) and the cells irradiated with 0.1 J/cm^2^ (*p* = 0.0001), 0.2 J/cm^2^ (*p* = 0.0003) and 1.2 J/cm^2^ (*p* = 0.026) compared to the cells irradiated with 10 J/cm^2^. A significant diminution of the surface area was also noted between the control positive (*p* < 0.0001) and the cells irradiated with the two lower energy doses (*p* < 0.01) compared to the control negative, and between the control positive and the cells irradiated with 1.2 J/cm^2^ (*p* = 0.0013). There was no significant difference in the linear mean length between the control negative and the cell irradiated with 1.2 J/cm^2^ (*p* = 0.2358), nor between the three groups with lower irradiation (*p* > 0.08). ^a,b,c,d^ Values with different letters are significantly (*P* < 0.05) different

**Fig. 7:**
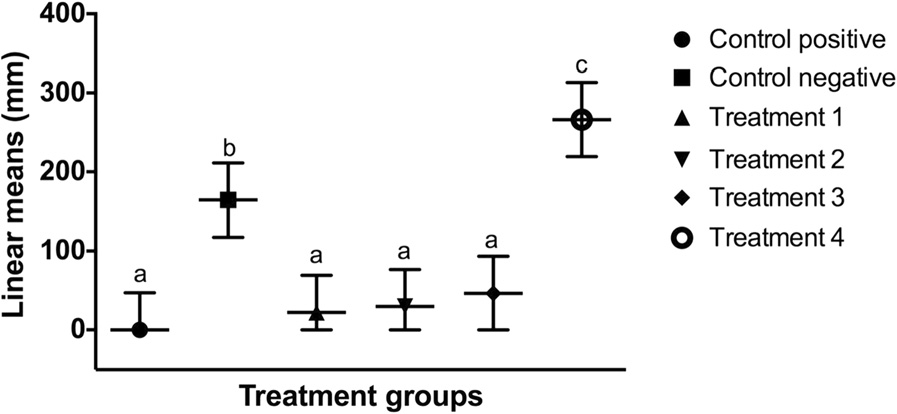
Effect of LLLT on wound size 24 h after irradiation. Linear mean length with the confidence interval for each wounded area is presented. The linear mean length of the control positive and the cells irradiated with 0.1, 0.2, and 1.2 J/cm^2^ were significantly decreased compared to the two other groups (*p* < 0.0005). There was also a significant difference noted in the linear mean length of the control negative compared to the cells irradiated with 10 J/cm^2^ (*p* = 0.0026). ^a,b^ Values with different letters are significantly (*P* < 0.05) different

**Fig. 8:**
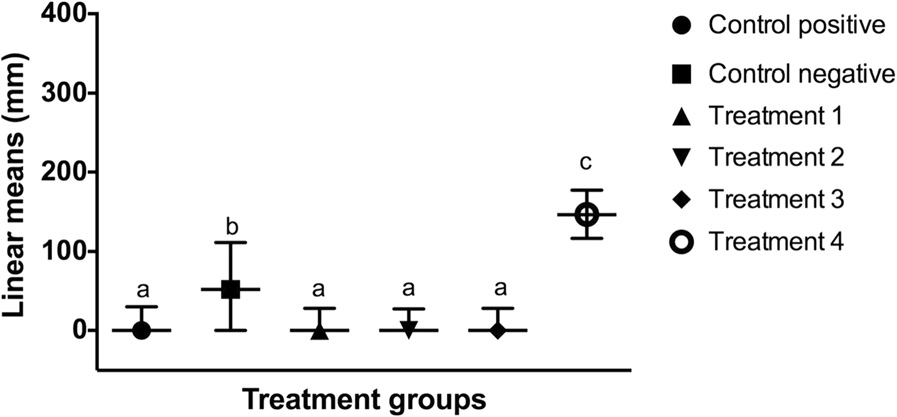
Effect of LLLT on wound size 36 h after irradiation. Linear mean length with the confidence interval for each wounded area is presented. As for the 24-h time point results, there was no significant difference in the linear mean length between the control positive and the three lower energy density groups (p > 0.5). The linear mean length of the control positive and the cells irradiated with 0.1, 0.2, and 1.2 J/cm^2^ was significantly decreased compared to the two other groups (*p* < 0.03). There was also a significant difference noted in the linear mean of the control negative compared to the cells irradiated with 10 J/cm^2^ (*p* < 0.0001). ^a,b^ Values with different letters are significantly (*P* < 0.05) different

The linear mean length of each wounded region of the cell monolayer was compared with control groups and with each other over time. There was no statistical difference (*p* > 0.05) in the linear mean length of each scratch created in the cell monolayer at the beginning of the study period (Fig. 5). Twelve hours following LLLT (Fig. 6), there was a significant reduction in the linear mean length of the control positive (*p* < 0.0001) and the cells irradiated with 0.1 J/cm^2^ (*p* = 0.0001), 0.2 J/cm^2^ (*p* = 0.0003) and 1.2 J/cm^2^ (p = 0.026) compared to the cells irradiated with 10 J/cm^2^. A significant diminution of the area was also noted between the control positive (*p* < 0.0001) and the cells irradiated with the two lower energy doses (*p* < 0.01) compared to the control negative, and between the control positive and the cells irradiated with 1.2 J/cm^2^ (*p* = 0.0013). Twenty-four hours (Fig. 7) and 36 h (Fig. 8) following LLLT, there was no significant difference observed in the linear mean length of the control positive wounded region compared to the groups irradiated with 0.1, 0.2, and 1.2 J/cm^2^ (*p* > 0.15). The positive control and all three groups with lower irradiation did, however, show a significantly reduced linear mean length compared to the control negative (*p* < 0.03) and the group irradiated with 10 J/cm^2^ (*p* < 0.0001). A significant difference was also identified between the control negative and the cells irradiated with 10 J/cm^2^ (*p* < 0.003) at both time points.

For all 15 experiments, the time of closure among groups was similar and followed a predictable pattern. The cells irradiated with the highest dose of energy (10 J/cm^2^) reliably showed the slowest rate of closure compared to all other groups. Overall, the results of the scratch migration assay revealed that wounded canine epidermal keratinocytes closed earlier in the non-irradiated cultures maintained in 10 % serum medium (positive control) and in cultures exposed to a single dose of 0.1, 0.2 or 1.2 J/cm^2^ compared to the non-irradiated cells cultured in serum-reduced medium (negative control) and the cells irradiated with 10 J/cm^2^.

### Proliferation Assay

A total of fifteen experiments, from cell passages three to five, were included in the study. Three replicated plates were used for each experiment to evaluate cellular proliferation over time. An outlier was identified in the proliferation assay but was retained in the analysis. The assumption of normality was mildly violated because of that one outlier; otherwise the assumption of the ANOVA analysis was adequately met. No transformation was required.

**Fig. 9:**
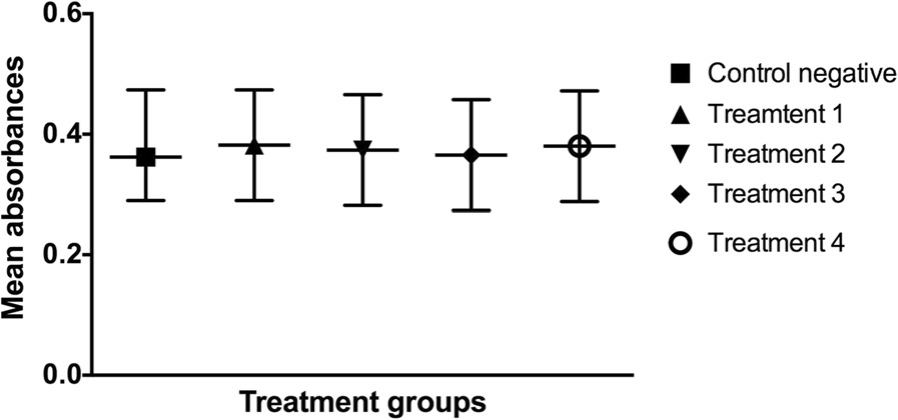
Effect of LLLT on cellular proliferation immediately after irradiation. Mean absorbance with the confidence interval for each group is presented. There was no statistical difference noted at the beginning of the experiment (*p* > 0.5). Significance *p* < 0.05

**Fig. 10:**
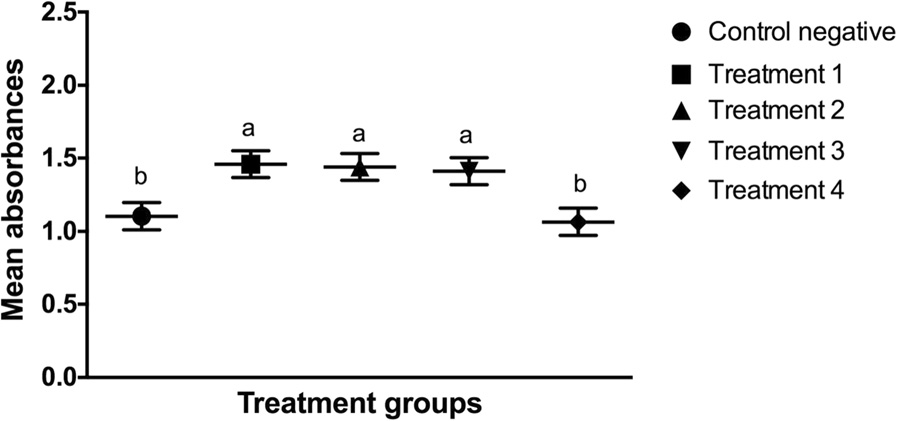
Effect of LLLT on cellular proliferation 24 h after irradiation. Mean absorbance with the confidence interval for each group is presented. The mean absorbance of the cells treated with 0.1, 0.2 and 1.2 J/cm^2^ was significantly increased compared to the other groups (*p* < 0.0001). There was no significant difference between the non-irradiated cells cultured in serum-deprived medium and the cells irradiated with 10 J/cm^2^ at that time point (*p* = 0.3642). ^a,b^ Values with different letters are significantly (*P* < 0.05) different

**Fig. 11:**
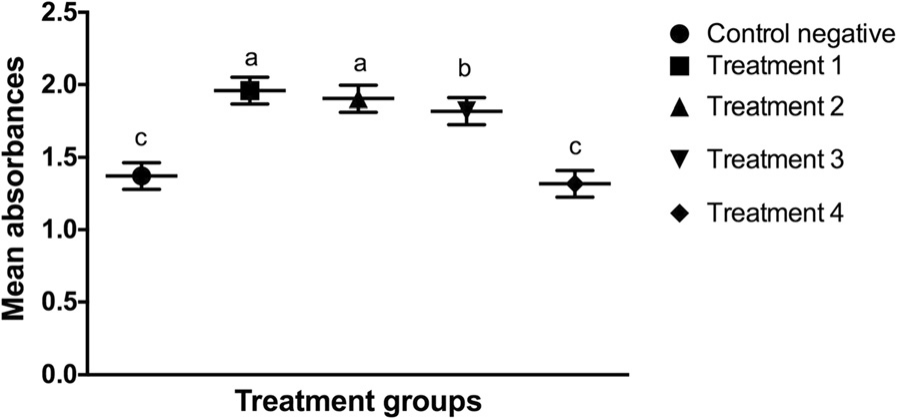
Effect of LLLT on cellular proliferation 48 h after irradiation. Mean absorbance with the confidence interval for each group is presented. The mean absorbance of the cells treated with 0.1, 0.2 and 1.2 J/cm^2^ was significantly increased compared to the other groups (*p* <0.0001). The cells irradiated with 0.1 J/cm^2^ (*p* = 0.01) and 0.2 J/cm^2^ (*p* = 0.0434) had significantly greater cellular proliferation compared to the cells irradiated with 1.2 J/cm^2^. There was no significant difference in the cellular proliferation rate between the two lower energy doses (*p* = 0.1987), or between the control group and the cells irradiated with 10 J/cm^2^ (*p* = 0.2011). ^a,b,c^ Values with different letters are significantly (*P* < 0.05) different

The number of cells at each time point was assessed by comparing the mean absorbance of the formazan dye produced by cellular metabolism following the addition of the cell proliferation reagent WST-1. There was no statistical difference (*p* > 0.5) in the mean absorbance among groups at the beginning of the study period (Fig. 9). Twenty-four hours after LLLT, the mean absorbance of the cells irradiated with 0.1, 0.2, and 1.2 J/cm^2^ was significantly increased compared to the non-irradiated cells cultured in serum-deprived medium (*p* < 0.0001) and the cells irradiated with 10 J/cm^2^ (*p* < 0.0001). There was no significant difference between the control group and the cells irradiated with 10 J/cm^2^ at that time point (*p* = 0.3642) (Fig. 10). Forty-eight hours after LLLT, the mean absorbance of the treatment groups irradiated with 0.1, 0.2, and 1.2 J/cm^2^ was significantly higher than the control cultures (*p* < 0.0001) and the cultures irradiated with 10 J/cm^2^ (*p* < 0.0001). In addition, the treatment groups irradiated with 0.1 J/cm^2^ (p = 0.001) and 0.2 J/cm^2^ (*p* = 0.0434) had significantly greater cellular proliferation compared to the cells irradiated with 1.2 J/cm^2^. There were no significant differences in cellular proliferation between the two lower energy doses (*p* = 0.1987), or between the control negative group and the cells irradiated with 10 J/cm^2^ (*p* = 0.2011) at the 48-h time point (Fig. 11).

The results of the cell proliferation assay showed that canine epidermal keratinocytes exposed to a single dose of 0.1, 0.2 and 1.2 J/cm^2^ proliferated more rapidly than the non-irradiated cells cultured under the same serum-reduced conditions, and the cells irradiated with 10 J/cm^2^.

## Discussion

The present study has evaluated the effects of LLLT on in vitro responses in a model of wound healing using cultured canine epidermal keratinocytes. To the authors’ knowledge, this project is the first of its kind in veterinary medicine. Low-level laser therapy was delivered as a single exposure from a high-power He-Ne Class IV laser unit system (650 nm). Cellular migration and proliferation, which are both essential in the normal wound healing process in vivo, were evaluated.

The scratch migration and cell proliferation WST-1 assays have both been employed frequently in other wound healing experiments in the literature [18–20]. The scratch migration assay was selected for this study, as it is a straightforward, relatively inexpensive, reproducible, and well-defined method described in the literature to quantify cellular migration parameters [18]. The scratch migration assay represents a widely accepted method to study cellular migration in vitro, and it models many aspects of in vivo cellular migration [19]. To evaluate and compare cellular proliferation among all groups over time, the ready-to-use cell proliferation reagent WST-1 was employed. This reagent provides a simple and accurate method to measure cellular metabolic activity, which is based on the cleavage of the tetrazolium salt by cellular mitochondrial dehydrogenases to soluble, non-cytotoxic, highly colored end product, called formazans [21]. An increase in the number of viable cells results in higher mitochondrial dehydrogenase activity (mitochondrial numbers are relatively constant on a per cell basis), which leads to a greater amount of formazan dye production [21]. The formazan dye is then quantified by measuring the light absorbance at the wavelength of maximal absorbance (450 nm) [21]. The quantity of formazan dye produced is directly proportional to the number of metabolically active cells in the culture medium [21]. The method is considered more rapid and more sensitive than those using other tetrazolium compounds—MTT-, XTT-, or MTS [22].

The results of the scratch migration and proliferation assays showed that canine epidermal keratinocytes produce a measurable biological response in vitro that is likely favorable for wound healing when exposed to low doses of LLLT (0.1, 0.2 and 1.2 J/cm^2)^, compared to non-irradiated cell cultures also maintained in serum-deprived medium. Overall, the positive control group in which rich-serum medium—containing numerous additional growth factors and nutrients—showed the fastest rate of closure, which was expected. This rate of closure was, however, not significantly different when compared to the cells maintained in serum-deprived medium and exposed to low-levels of laser therapy. Because a cell proliferation reagent was added directly to the medium in the cell proliferation assay, a positive control was not included as the metabolic consequences of the high serum concentration that is present alters the spectral properties of the medium, making absorbance analysis invalid between samples that contain hight serum concentration and those that do not. Significant stimulatory effects of LLLT on cellular migration and proliferation of canine keratinocytes was observed. These results are in agreement with other experimental analyses that report a beneficial effect of LLLT in wound healing and help identify the individual processes that are influenced in keratinocytes [3–13].

Importantly, LLLT at the highest exposure dosage (10 J/cm^2^) resulted in an inhibitory effect, suggesting that dose level is extremely important in the overall biologic response. Dose-responses of this type have been previously described in several *in vivo* clinical experiments, animal models, and cell cultures [12, 23, 24]. The Arndt-Schulz Law is commonly cited as an appropriate model to demonstrate the dose-dependent effect of LLLT and suggests that low levels of light have a better effect in wound healing than higher levels, which may have an inhibitory or cytotoxic effect [23, 24]. In their work to establish the behavior in vitro of human skin fibroblasts, Hawkins and Abrahamse (2006) used a He-Ne laser (632.8 nm) at different irradiation doses of 0.5, 2.5, 5, 10, and 16 J/cm^2^. They demonstrated that higher laser doses (10 and 16 J/cm^2^) resulted in increased cellular damage as well as decreased cellular viability and proliferation [25]. Houreld and Abrahamse (2008) used He-Ne (632.8 nm), Gallium-Aluminum-Arsenide (GaAlAs (830 nm)), and Neodymium Yttrium Aluminum Garnet (1064 nm) lasers for treatment of in vitro wounded diabetic-induced fibroblasts irradiated with either 5 or 16 J/cm^2^. Regardless of the wavelength used, all cells irradiated with 16 J/cm^2^ showed incomplete wound closure, increased apoptosis, and decreased basic fibroblast growth factor expression. The fibroblasts responded better overall when irradiated with an energy density of 5 J/cm^2^ at a wavelength of 632.8 nm [26]. Similarly, Basso et al. (2012) demonstrated that irradiation with Indium Gallium Arsenide Phosphide diode laser (780 nm) of cultured human gingival fibroblasts with energy doses of 0.5 and 3 J/cm^2^ resulted in a significant increase in cellular metabolism compared with the non-irradiated control group and the cells irradiated with higher energy doses of 5 and 7 J/cm^2^ [27]. Limited animal model studies have also shown similar dose-dependent responses, leading to discrepancies in clinical outcomes observed. In their work on wound healing, Demidova-Rice and collaborators (2007) documented this phenomenon when they tested the effect of 635 nm non-coherent light at 1, 2, 10 and 50 J/cm^2^ on full-thickness dorsal excisional wounds in mice. Comparison of the area under the healing curves generated based on the wound healing rates over time among groups revealed that a single exposure to 1, 2 and 10 J/cm^2^ improved wound healing compared to the non-irradiated control group, while the group irradiated with 50 J/cm^2^ had an inhibitory effect [12]. The concept of biphasic dose-response is important in LLLT and the complexity of choosing amongst a large number of illumination parameters for each treatment may explain why there is disagreement among clinical results and lack of clear conclusions regarding the observed effects of this therapeutic modality [23–27]. In addition, Karu (1989) proposed that the magnitude of the laser photostimulation effect was dependent of the physiological state of the cell at the time of irradiation. Stressed, damaged, or poorly growing cultures respond overall better to the positive effect of laser therapy [28]. Karu’s statement may also explain why the effect is not always detectable and why there are conflicting results found in the literature. Similar to other cell culture studies, the present analysis demonstrated that canine epidermal keratinocyte cultures irradiated with the highest dose (10 J/cm^2^) had the slowest in vitro wound closure rate and proliferation rate compared to cells irradiated with lower energy doses of 0.1, 0.2, and 1.2 J/cm^2^. Overall, these results clearly show that LLLT can stimulate or impede cellular processes in a dose-dependent manner.

Low-level laser therapy is believed to enhance wound healing and to provide pain relief through a variety of cellular processes [3–6]. Low-level laser therapy has recently been shown to have an effect on micro-RNA (MiRNA) expression [14, 15]. However, the exact mechanisms by which LLLT influences cellular processes remain uncertain and is likely multifactorial [4, 5, 10–12]. At the cellular level, the most commonly accepted theory to explain the effects of LLLT is thought to be absorption of red and near infrared light by the photoreceptor cytochrome c oxidase located in mitochondria [11, 12]. Few experiments have shown that cellular respiration is increased when mitochondria are exposed to light in the red and near-infrared spectral range [29–31]. Of them, Chen *et al.* (2008) demonstrated, in their study on endothelial cells, that low-energy laser irradiation at the cellular level increases endothelial cell proliferation, migration, and endothelial nitric oxidase synthetase (eNOS) protein expression through activation of the PI3K/Akt pathway [29]. Huang and colleagues (2013) provided evidence that low-power laser irradiation using a HeNe laser at a wavelength of 633 nm and dose of 1.2 J/cm^2^ induced nuclear redistribution and transcriptional activity of estrogen receptors, through activation of the PI3K/Akt signaling cascade, which may control cellular processes and regulation of gene expression [30]. Silveira *et al.* (2006) successfully demonstrated by mitochondrial enzyme evaluation that red and near-infrared light delivered for 10 days to iatrogenic wounds created on adult male Wistar rats significantly increased the activities of the respiratory chain enzyme complexes II and IV (cytochrome c oxidase). The instrument used in this study was a GaAlAs laser with a wavelength of 904 nm [31]. The results of these analyses are in agreement with other data from the literature where cytochrome c oxidase is thought to be an important photoreceptor of light in the red to near-infrared spectrum [11–13, 29–31]. However, more research is required to fully evaluate mitochondrial enzyme activities following LLLT and to understand its mechanisms of action at the cellular level.

## Conclusion

In conclusion, this study has provided significant findings using a previously-untested cell type concerning the biological effects of LLLT on important cellular parameters associated with wound healing in cultured canine epidermal keratinocytes. Cell culture models are useful tools to evaluate the efficacy and safety of LLLT and are strongly indicated prior to the application of this treatment modality in a clinical setting. The results of this study demonstrated that low-levels of laser irradiation delivered as a single dose caused increased migration and proliferation of canine epidermal keratinocytes compared to the control negative group. Low-level laser therapy may stimulate or impede cellular processes in a dose-dependent manner, suggesting that appropriate protocol selection will be critical for improved clinical outcomes. Further in vitro and in vivo studies will be required to fully investigate the effects of LLLT on canine wound healing and the mechanisms by which this treatment mediates its effects so that continued improvements can be made.

## Abbreviations

CPEK: Canine epidermal keratinocyte progenitors
He-Ne: Helium-Neon
LLLT: Low-level laser therapy
RCBD: Randomized complete block design
WST-1: Water-soluble tetrazolium

## Footnotes

1 CELLnTEC Advance Cell Systems AG, Bern, Switzerland

2 Fisher Scientific, Ottawa, Ontario, Canada

3 Sigma, St. Louis, Missouri

4 VWR, Radnor, Philadelphia

5 Olympus Canada Inc., Richmond Hill, Ontario, Canada

6 CTS Therapy System, Companion Therapy Laser, LiteCure LCC, Newark, Delaware

7 IX81, Olympus America Inc., Melville, New York

8 Adobe Systems Incorporated, New York, New York

9 Roche, Mannheim, Germany

10 Fisher Scientific, Ottawa, Ontario, Canada

11 Fluostar Optima, BMG Lab-technologies, Offenburg, Germany

12 Proc MIXED, SAS Institute Inc., Cary, North Carolina
